# Percutaneous Endoscopic Interbody Debridement and Fusion (PEIDF) Decreases Risk of Sepsis and Mortality in Treating Infectious Spondylodiscitis for Patients with Poor Physical Status, a Retrospective Cohort Study

**DOI:** 10.3390/biomedicines10071659

**Published:** 2022-07-10

**Authors:** Sheng-Fen Wang, Tsung-Ting Tsai, Yun-Da Li, Ping-Yeh Chiu, Ming-Kai Hsieh, Jen-Chung Liao, Po-Liang Lai, Fu-Cheng Kao

**Affiliations:** 1 Department of Anesthesiology, Chang Gung Memorial Hospital, College of Medicine, Chang Gung University, Taoyuan 333, Taiwan; wangfenn@gmail.com; 2 Department of Orthopaedic Surgery, Spine Section, Bone and Joint Research Center, Chang Gung Memorial Hospital, College of Medicine, Chang Gung University, No. 5, Fusing St., Gueishan, Taoyuan 333, Taiwan; tsai1129@gmail.com (T.-T.T.); yunda1222@hotmail.com (Y.-D.L.); jobphage@cgmh.org.tw (P.-Y.C.); mk660628@gmail.com (M.-K.H.); johnliao1002@gmail.com (J.-C.L.); polianglai@gmail.com (P.-L.L.); 3 Institute of Biomedical Engineering, National Tsing Hua University, Hsinchu 300, Taiwan

**Keywords:** sepsis, qSOFA, infectious spondylodiscitis, endoscopy

## Abstract

**Background:** Postoperative immunosuppression is associated with blood loss and surgical trauma during surgery and subsequently predisposes patients to increased morbidity. Spine endoscopic surgery has been accepted as an effective surgical technique with less surgical trauma and less blood loss for the complication of infectious spondylodiscitis. Therefore, the aim of this study was to investigate whether PEIDF could reduce the morbidity rates for patients with infectious spondylodiscitis. **Methods:** We launched a retrospective cohort study on the comparison of the perioperative prognosis between PEIDF and conventional open surgery for single-level lumbar infectious spondylodiscitis in patients with poor physical health (ASA ≥ 4) from 2014 to 2019. **Results:** Forty-four patients were included in this study. Fifteen of them underwent PEIDF, and the rest of the 29 patients were treated with open surgery. Less surgical blood loss (*p* < 0.001) and intraoperative transfusions (*p* < 0.001) with a better decline of CRP (*p* = 0.017) were statistically significant in patients receiving PEIDF. Patients undergoing conventional open surgery encountered more postoperative sepsis (*p* = 0.030), a higher qSOFA score (*p* = 0.044), and prolonged-time for CRP normalization (*p* = 0.001). **Conclusions:** PEIDF minimizes a poor postoperative outcome due to less surgical trauma, intraoperative blood loss, and the need for a blood transfusion.

## 1. Introduction 

Postoperative sepsis is one of the major complications in spine surgery, which is highly associated with organ dysfunction and climbing morbidity and mortality [1]. For patients developing sepsis, it reaches up to a 40–60% chance of progressing to septic shock and 25–30% to mortality [2]. The post-surgical immunosuppression is considered one of the critical factors predisposing patients to life-threatening sepsis [3]. Intraoperative blood loss and transfusion [4], reperfusion injury, the extent of surgical trauma, and surgical stress all have an impact on postoperative immune system dysfunction. In addition, patients’ physical status, underlying conditions, and factors, such as age or gender, also play a pivotal role in immunomodulation [5]. 

Compared with open surgery, endoscopic spine surgery has been accepted as an effective surgical technique with minimal invasion, less surgical trauma, less blood loss, and better recovery, especially for patients with a poor physical status [6,7,8]. Many patients with infectious spondylodiscitis are immunocompromised with multiple comorbidities and assumed as poor candidates for open surgery. Percutaneous endoscopic techniques used in infectious spondylodiscitis have been proven to prevent surgery-related complications, including massive intraoperative bleeding, surgical tissue damage, postoperative hemorrhage, and further disability [8]. Percutaneous endoscopic interbody debridement and fusion (PEIDF) is a relatively new surgical technique that we proposed for the treatment of infectious spondylodiscitis to eradicate infectious foci and restore spinal stability [9]. The post-surgical immunosuppression might benefit from PEIDF due to decreased intraoperative blood loss, blood transfusion, surgical trauma, and surgical stress, thus lowering the risk of postoperative sepsis and a poor outcome. 

Therefore, the aim of this retrospective cohort study was to compare the safety and efficiency of PEIDF to the conventional open approach and investigate whether PEIDF could reduce the rate of postoperative sepsis and poor outcomes for patients with infectious spondylodiscitis and poor physical health (American Society of Anesthesiologists (ASA) physical status class 4 or higher). 

## 2. Materials and Methods

### 2.1. Study Population

We launched a retrospective cohort study using the database from the orthopedic department of our institution. From April 2014 to July 2019, all patients receiving surgical treatment for infectious spondylodiscitis were carefully recruited and surveyed. The exact diagnosis was preoperatively confirmed based on clinical symptoms, elevated inflammatory and biochemical markers, and image results. In this study, we selected the cohort as patients with poor physical health [10] (ASA ≥ 4) receiving surgical treatment due to single-level lumbar infectious spondylodiscitis and focused on the comparison of the perioperative prognosis between PEIDF and conventional open surgery. Due to the nature of the retrospective study, the type of procedure was decided by the surgeon’s preference after a thorough preoperative discussion and explanation with patients. Surgical indications for infectious spondylodiscitis included intractable back pain, failure to antibiotic treatment for at least 3–4 weeks, spinal instability from severe destruction, progressive neurological symptoms, impending systemic organ failure, and hemodynamic instability. Patients undergoing cardiopulmonary resuscitation (CPR) during operation, suffering from multiple major infectious foci, and multilevel spine infection were excluded. 

The patients were under careful treatment after surgery either in the orthopedic ward or intensive care unit, depending on their general postoperative condition. Continuous intravenous antibiotics were prescribed based on the culture reports and suggestions from the infectious disease specialists for 3–4 weeks, adjusted according to the improvement of inflammatory markers, and then followed by oral antibiotics until a minimum of 3 months of total antibiotic therapy (intravenous and oral antibiotics). All patients were encouraged to sit and ambulate with a brace after surgery if their physical condition permitted. 

This study had institutional review board approval, and the need to obtain informed patient consent was waived due to the retrospective nature of the study. All procedures performed in this study complied with the ethical standards of the national research committee. The data of patients were anonymized and maintained confidentiality. 

### 2.2. Outcome Measurement and Data Collection

The primary outcome of this study was assessed by episodes and the time-to-event data of sepsis, a quick Sequential Organ Failure Assessment (qSOFA) with a score [11] of ≥2, and death within one month after the operation. The secondary outcome was the time of the normal C-reactive protein (CRP) level. The following variables were retrieved and analyzed from the selected patients: age, gender, comorbidity, laboratory data, culture data, image data, surgical type, surgical time, back pain VAS score (Visual Analogue Scale), intraoperative blood loss, and transfusion. 

### 2.3. Surgical Techniques

#### 2.3.1. PEIDF

**Figure 1:**
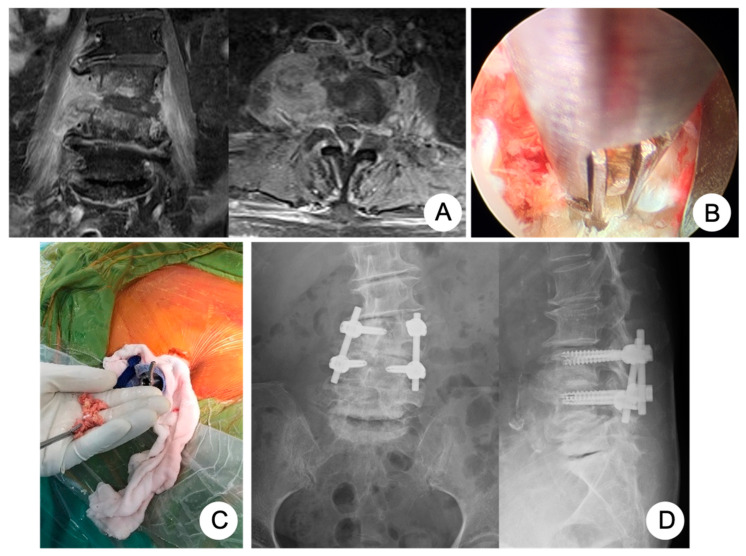
An 81-year-old female had the comorbidity of chronic kidney disease, stage 4, caused by poor-controlled diabetes mellitus, hypertension with atrial fibrillation, and ovarian cancer history, suffering from infectious spondylodiscitis complicated with severe endplate destruction and psoas muscle abscess (**A**). We performed PEIDF composed of percutaneous endoscopic debridement (**B**) and allogenous bone grafting (**C**) through the working sheath and posterior percutaneous pedicle screw fixation at one level above and below under the guidance of fluoroscopy (**D**).

The PEIDF was composed of a two-part procedure: a percutaneous endoscopic debridement plus bone-grafting interbody fusion and percutaneous posterior instrumentation. First of all, multi-directional debridement and discectomy would be performed right after the working sheath was inserted into the target infected disc from the posterolateral side of the waist, under the guidance of fluoroscopy (Figure 1B). Following radical debridement and a large amount of normal saline irrigation of the infected disc, the allogenous bone chips were impacted into the disc space through the working sheath for interbody fusion (Figure 1C). The second part was a posterior percutaneous screw fixation at one level above and below (Figure 1D) under the guidance of fluoroscopy, avoiding back muscle dissection and soft tissue trauma. The details of the PEIDF are well described in our previous study [9]. 

#### 2.3.2. Posterior Open Surgery

One posterior midline approach was made for the exposure of anatomic structures. Posterior instrumentation was performed first by inserting trans-pedicle screws into the vertebrae 1 or 2 levels above and below the lesion, according to the stability and quality of the bone. Then, radical discectomy and debridement of the infected disc would be made by the TLIF (transforaminal lumbar interbody fusion) technique through unilateral facetectomy. Allogenous bone grafts were inserted and compressed into the disc space for interbody fusion. 

#### 2.3.3. Anterior Open Surgery

The patients underwent an anterolaterally retroperitoneal approach in the lateral decubitus position with the left side up. Anterior discectomy and radical debridement were performed directly to eradicate the infected tissue, and allogenous cortical struts were compacted into the disc space for interbody fusion. 

#### 2.3.4. Statistical Analysis

Statistical calculations were performed by SPSS (version 27; SPSS Inc., Chicago, IL, USA). Quantitative variables were presented as mean ± standard deviations. Continuous variables were analyzed by the Student’s *t*-test. The categorical variables were assessed by the Chi-square test. The analysis of the time-to-event data in the case of sepsis, a qSOFA of ≥2, and death within the postoperative one month was accessed by the Kaplan–Meier method and the log-rank test was used to compare the differences between the two groups. A *p*-value  of <0.05 was considered to be a significant difference.

## 3. Results

### 3.1. Study Objects

**Figure 2:**
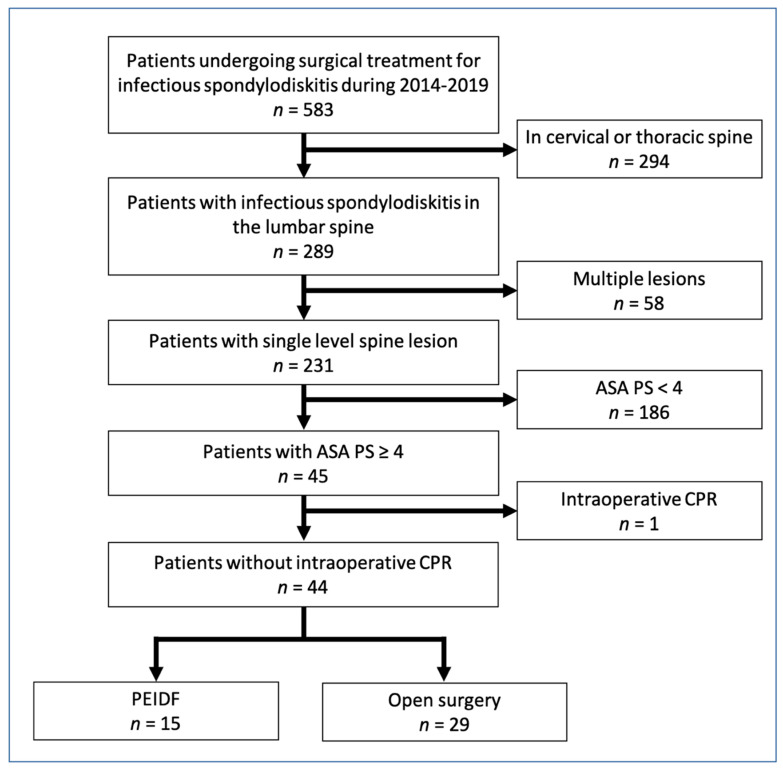
Flowchart of 2014–2019 cohort sample. ASA PS: American Society of Anesthesiologists physical status; CRP: C–reactive protein; PEIDF: percutaneous endoscopic interbody debridement and fusion.

From April 2014 to July 2019, a total of 289 patients with infectious spondylodiscitis in the lumbar spine were extracted from our database out of 583 patients (49.6%) undergoing surgical treatment for infectious spondylodiscitis. We further excluded multilevel spine lesions and multiple major infectious foci (*n* = 58), their ASA physical status < 4 (*n* = 186), and intraoperative CPR (*n* = 1). The remaining 44 patients receiving surgical treatment for single-level lumbar infectious spondylodiscitis were chosen as the cohort in this study. Fifteen of them underwent PEIDF, and the rest of the 29 patients were treated with conventional open surgery (six patients by the anterior approach and 23 patients by the posterior approach). Figure 2 illustrates a detailed flow diagram of the patients enrolled in the final analysis.

### 3.2. Demographic and Clinical Data

The demographic and clinical results of the analysis between PEIDF and open surgery are demonstrated in Table 1. There was no significant difference in patients’ age, gender, comorbidities, pyogenic burden (psoas and epidural abscess), preoperative laboratory data (albumin, hemoglobin, CRP level, white blood cell (WBC) count, segment percentage, and platelet), and intraoperative culture rate. However, intraoperative blood loss and transfusion were significantly less in patients receiving PEIDF even under a similar surgical time. For the postoperative laboratory data, the hemoglobin levels of the postoperative day one showed discrepancy, yet without a significant difference (*p* = 0.059). The postoperative-one-month CRP values revealed better improvement in PEIDF with statistical significance (*p* = 0.017), while other postoperative data showed no difference. In addition, patients receiving PEIDF experienced better pain relief after the postoperative one week (*p* < 0.001), even though all of the patients had a similar back pain intensity preoperatively. 

### 3.3. Outcome Assessment

**Figure 3:**
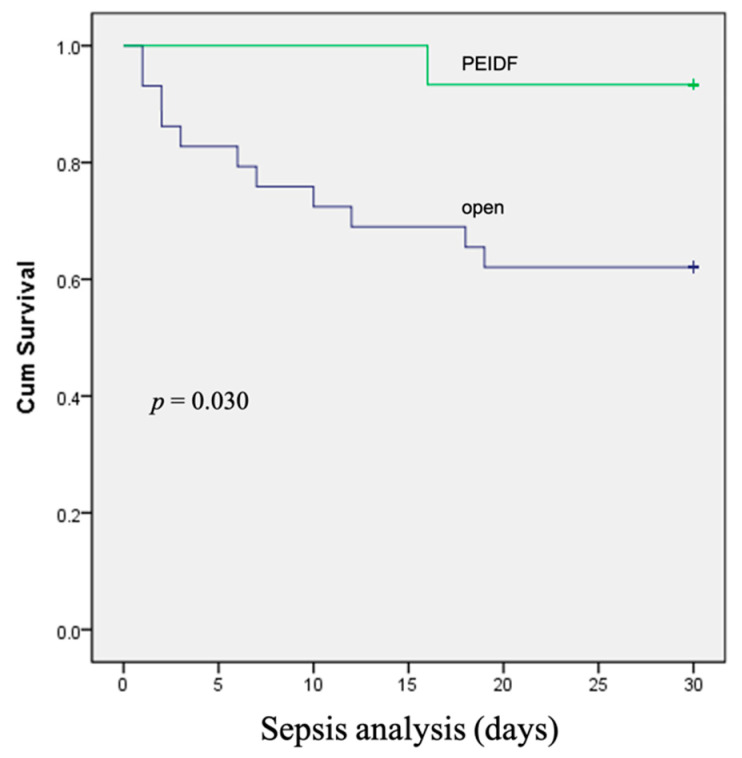
The Kaplan–Meier curve shows that patients receiving conventional open surgery had a higher rate of sepsis within one month after surgery.

**Figure 4:**
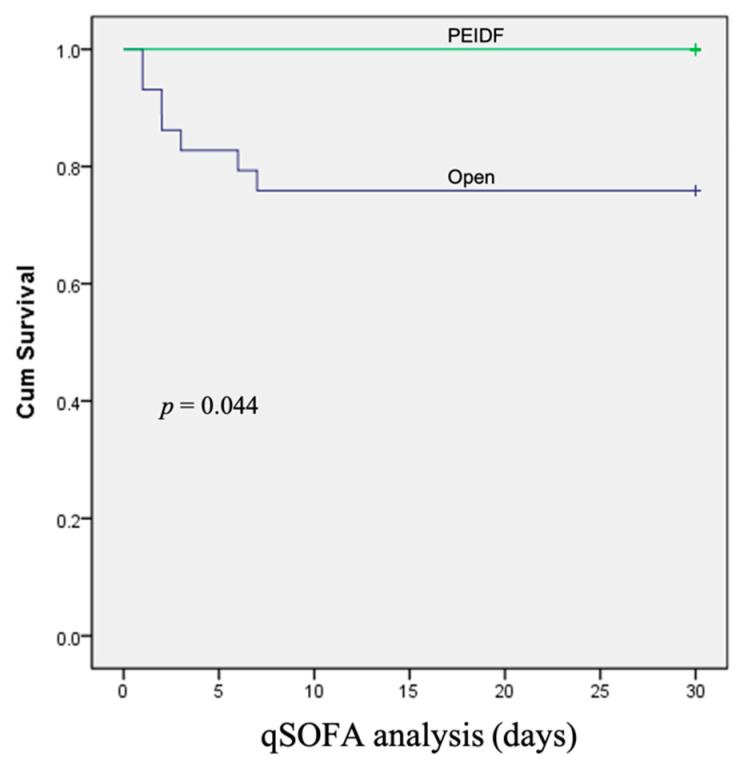
The Kaplan–Meier curve shows that patients receiving conventional open surgery had a higher rate of a qSOFA of ≥2 within one month after surgery.

**Figure 5:**
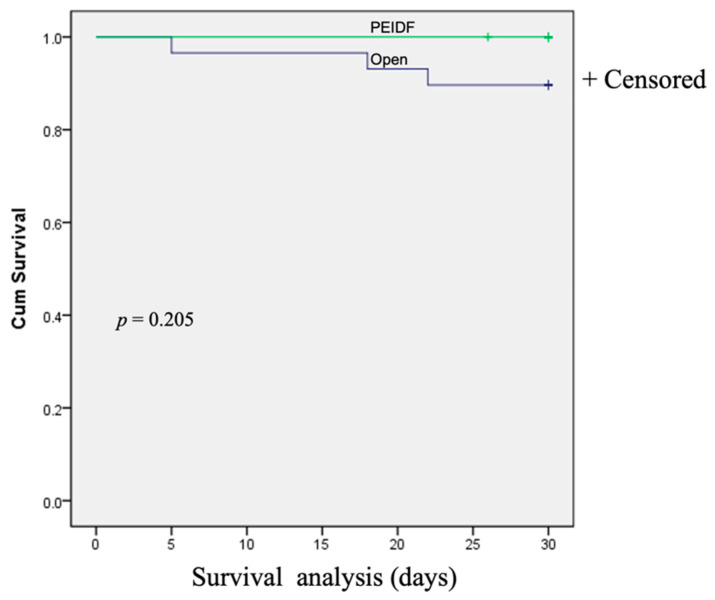
The Kaplan–Meier survival curve demonstrates the postoperative mortality within one month between the two groups.

No patient has suffered from intraoperative neurological injury or postoperative epidural hematoma compression in this study. Two patients receiving PEIDF had unsatisfactory outcomes: one patient with end-stage renal disease suffered from an uneven decline of CRP postoperatively and experienced a sepsis episode during hospitalization; the other patient with a preoperative traumatic subdural hemorrhage encountered a sudden onset subarachnoid hemorrhage in the intensive care unit about one month postoperatively. The primary outcome of this study was evaluated by sepsis, a qSOFA of ≥2, and death within one month after the operation. One patient undergoing PEIDF had sepsis within one month postoperatively, compared with 11 of the open surgery patients (6.7% versus 37.9%, *p* = 0.030). A high qSOFA score (more or equal to 2) was found in seven patients within one month after the open surgery, which was significantly higher than the PEIDF group (24.1% versus 0%, *p* = 0.044). Three patients died from septic shock after the open surgery, and one patient died from a sudden onset subarachnoid hemorrhage in the PEIDF group (10.3% versus 6.7%, *p* = 0.205). No statistical significance was found in the mortality rate. The secondary outcome was the time for the normalization of CRP. We defined the normal value of CRP as less than 10 mg/L in this study. The patients receiving PEIDF took significantly shorter days (26.93) than those of open surgery (41.31) to reach CRP normalization (*p* = 0.001). The detailed results of the outcomes are shown in Table 2 and Figure 3, Figure 4 and Figure 5. 

## 4. Discussion

Compared with conventional open surgery, minimally invasive endoscopic surgery has been generally accepted as an alternative treatment for infectious spondylodiscitis with less surgical trauma and blood loss [12]. Tissue damage from surgical trauma and intraoperative blood loss is considered to be associated with a temporary status of postoperative immunosuppression. As the tissue damage and blood loss become excessive, the postoperative immunosuppression becomes more severe and apparent, increasing the risk of susceptibility to sepsis and septic shock [13]. According to the results of this study, we found that PEIDF significantly reduced intraoperative blood loss and the amount of blood transfusion, demonstrating a lower risk of postoperative sepsis and a high qSOFA score with better infection treatment and recovery compared with conventional open surgery. Therefore, we speculated three possible mechanisms that might be responsible for more preferable outcomes of PEIDF as the following: 

Above all, reduced blood loss and surgical trauma of PEIDF during operation would directly have fewer effects on the postoperative physical status. In clinical practice, many studies have demonstrated the advantages of minimal invasion and less blood loss in the aspects of a fast recovery and immune function after surgery. Friederike et al. reported similar study results as ours, showing that higher intraoperative blood loss increased the risk of postoperative spinal implant infection [14]. A. A. F. A. Veenhof et al. showed that patients receiving laparoscopy had accelerated postoperative recovery and a lower inflammatory response compared with open surgery [15]. In conventional open surgery, a larger amount of blood loss could lead to the susceptibility of systemic hypotension and compromised oxygen delivery to vital organs. Acute metabolic stress would be triggered and respond to the intensity of the surgical trauma and tissue hypoxia. This stress response could activate the hypothalamic-pituitary-adrenal axis and the sympathetic nervous system [16]. The activation of the hypothalamic-pituitary-adrenal axis would cause the release of glucocorticoids, resulting in lymphocytopenia and the upregulation of anti-inflammatory gene products [17]. The sympathetic nervous system promotes the exocytosis of catecholamine-filled vesicles into the bloodstream, leading to the inhibition of T-cell proliferation and NK cell cytotoxicity [18]. Both of the above would be greatly responsible for postoperative immunosuppression, predisposing patients to postoperative sepsis and septic shock. In addition, a significant loss of plasma constituents and leukocytes would also cause immune dysfunction and impair the nutritional status [19]. 

Furthermore, blood transfusion is considered an independent risk factor for infection and postoperative sepsis [20]. Although the exact mechanism is still not well established, transfusion-related immunomodulation may play an important role in immunosuppression after surgery [21]. One theory is about the circulating non-transferrin-bound iron (NTBI) from erythrophagocytosis, followed by lysosomal catabolism of the storage-damaged erythrocytes. NTBI and magnificent erythrophagocytosis could induce some specific cytokines, leading to macrophage dysfunction, lysosome damage, and then cell death [22]. Decreased phagocytes would promote post-translational immunosuppression. Moreover, NTBI has the ability to enhance the virulence of pathogens [23]. Taken together, these outcomes increase susceptibility to sepsis [24]. Another is the upregulation of IL-10, IL-4, and TGF-beta due to allogenic transfusions. These cytokines could operate as anti-inflammatory medicators and downregulate cellular immunity, causing transient depression in the immune system [25]. Additionally, the infusion of allogenic leukocytes is associated with inappropriate inflammation and immune response, potentially contributing to immunosuppression. In this study, more intraoperative blood transfusion was found in conventional open surgery, accompanied by a higher rate of postoperative sepsis, a qSOFA of ≥2, and delayed normalization of CRP. 

Last but not least, the advantages of our PEIDF procedure for infectious spondylodiscitis also plays an important role in reduced postoperative immunosuppression and sepsis. It contains two major parts [9]. One is anterior endoscopic debridement and allogenic bone grafting; the other is posterior pedicle screw instrumentation. Percutaneous endoscopic surgery provides a clear and detailed view of the surgical fields and allows us to perform radical sequestrectomy and debridement of all of the necrotic disc tissue with minimal soft tissue damage and blood loss. There was no concern over posterior contamination due to the intact fascia and posterior longitudinal ligament, which acted as a natural barrier to prevent infection from spreading. Furthermore, intervertebral reconstruction via allogenic bone grafting and percutaneous posterior instrumentation would establish the structural stability to decrease postoperative back pain and the formation of infection-associated hematoma. Our results revealed significantly lower postoperative pain intensity in patients undergoing PEIDF. Percutaneous posterior instrumentation has been accepted as an effective procedure for providing secure stabilization with less surgical trauma in minimally invasive spine surgery [26,27,28]. For patients suffering from infectious spondylodiscitis, pure posterior stabilization without surgical debridement could largely diminish the intensity of back pain. According to the findings of the study by LA Nasto et al. [29], percutaneous posterior instrumentation was a safe and effective procedure associated with early ambulation, faster recovery, and lower pain scores. Meanwhile, it prevents deformity and improves the quality of life even without surgical debridement for infectious foci. Due to the minimized soft tissue damage of percutaneous posterior instrumentation, it has a minimal effect on the infection healing process. The authors suggested that percutaneous posterior instrumentation could be a reliable alternative approach compared to conservative treatments, such as TLSO (thoracolumbosacral orthosis), without concerns over implant contamination or infection. In summary, PEIDF minimizes surgical trauma, blood loss, the need for a blood transfusion, and provides equally radical debridement and structural stability as the conventional open surgery, and thus preventing postoperative sepsis. 

From the literature reviews, our study is the first to investigate the postoperative sepsis and qSOFA score between spine endoscopic and open surgery for patients with poor physical status. However, the current study does have some limitations, which call for caution in the interpretation of its results. First, this study is a retrospective design. Due to the limited case number of restricted inclusion criteria, the study sample size is small. Owing to the retrospective nature of the study, bias and confounders are difficult to control, and some essential patient data may not be available because the data collection relies on retrospect and written records. Then, the decision of the surgical type may lead to selection bias. However, to diminish the influence of selection bias, we set up the same inclusion and exclusion criteria, surgical indications, data collection methods, and outcome evaluation for all patients. Furthermore, our database is based on one single medical center, which reflects only the clinical practice we applied. In the future, a multicenter study should be performed to fully explore the risk reduction of postoperative complications in PEIDF, especially in patients with poor physical status. 

## 5. Conclusions

The diminished risk of sepsis and a high qSOFA score with a better decline of CRP levels were found after the PEIDF procedure among patients with poor physical health. PEIDF minimizes surgical trauma, intraoperative blood loss, and the need for a blood transfusion and could provide equally radical debridement and structural stability as conventional open surgery. Thus, a poor postoperative outcome can just as well be lessened.

## Figures and Tables

**Table 1:** Demographics and clinical data.

	PEIDF	Open	*p*-Value
Number of patients	15	29	
Age (year)	68.93 ± 14.09	70.83 ± 7.96	0.635
Sex (Male/Female)	8/7	12/17	0.450
Comorbidity			
Diabetic Mellitus	5	13	0.462
ESRD	3	14	0.092
COPD	5	8	0.692
Malignancy history	3	4	0.594
Pyogenic burden			
Psoas abscess	8	11	0.328
Epidural abscess	2	10	0.135
Surgical data			
Surgical time (minute)	120.80 ± 25.79	128.49 ± 39.95	0.506
Blood loss (mL)	<50	662.07 ± 489.47	<0.001 *
Intraoperative transfusion	0	16	<0.001 *
Preoperative laboratory data			
Albumin (g/dL)	2.89 ± 0.73	3.14 ± 0.78	0.345
CRP (mg/L)	137.31 ± 78.21	140.66 ± 69.87	0.885
WBC count (1000/μL)	9.85 ± 2.95	12.49 ± 6.27	0.065
Segment (%)	75.47 ± 9.02	78.55 ± 9.89	0.320
Platelet (1000/μL)	257.33 ± 126.34	310.10 ± 162.97	0.280
Hemoglobin (g/dL)	9.85 ± 1.07	9.27 ± 1.76	0.177
Postoperative laboratory data			
CRP (mg/L)	17.87 ± 19.96	41.73 ± 8.13	0.017 *
WBC count (1000/μL)	6.67 ± 2.56	7.12 ± 2.17	0.451
Segment (%)	61.13 ± 8.04	62.47 ± 10.39	0.665
Platelet (1000/μL)	266.20 ± 63.59	263.13 ± 82.41	0.901
D1 Hemoglobin (g/dL)	9.81 ± 1.22	9.03 ± 1.27	0.059
Culture rate (%)	12 (80.0%)	18 (62.1%)	0.226
Revision within one month	0	5	0.135
VAS score			
Preoperative	7.87 ± 1.06	7.66 ± 1.289	0.588
Postoperative one week	2.73 ± 0.704	4.52 ± 1.661	<0.001 *

ESRD, end-stage renal disease; COPD, chronic obstructive pulmonary disease; CRP, C-reactive protein; WBC, white blood cell; D1 Hemoglobin, hemoglobin on postoperative day 1; VAS, Visual Analogue Scale; *: *p* < 0.05, statistical significance.

**Table 2:** Outcome assessment.

	PEIDF	Open	*p*-Value
Sepsis (%)	1 (6.7)	11 (37.9)	0.030 *
qSOFA ≥ 2 (%)	0 (0.0)	7 (24.1)	0.044 *
Death (%)	1 (6.7)	3 (10.3)	0.205
Days to normal CRP level	26.93 ± 11.55	41.31 ± 12.32	0.001 *

qSOFA: quick sequential organ failure assessment. *: *p* < 0.05, statistical significance.

## Data Availability

The data presented in this study are available on request from the corresponding author. The data are not publicly available due to their containing information that could compromise the privacy of research participants.
